# Revealing underlying factors of absenteeism: A machine learning approach

**DOI:** 10.3389/fpsyg.2022.958748

**Published:** 2022-12-01

**Authors:** Francis Bowen, Carolyn Gentle-Genitty, Janaina Siegler, Marlin Jackson

**Affiliations:** ^1^Data Analytics and Business Intelligence Lacy School of Business, Butler University, Indianapolis, IN, United States; ^2^School of Social Work, Indiana University Bloomington, Bloomington, IN, United States; ^3^Fight for Life Foundation, Indianapolis, IN, United States

**Keywords:** absenteeism, school attendance problems, machine learning, socio-emotional learning, classification, multi-tiered systems of support

## Abstract

**Introduction:**

The basis of support is understanding. In machine learning, understanding happens through assimilated knowledge and is centered on six pillars: big data, data volume, value, variety, velocity, and veracity. This study analyzes school attendance problems (SAP), which encompasses its legal statutes, school codes, students’ attendance behaviors, and interventions in a school environment. The support pillars include attention to the physical classroom, school climate, and personal underlying factors impeding engagement, from which socio-emotional factors are often the primary drivers.

**Methods:**

This study asked the following research question: What can we learn about specific underlying factors of absenteeism using machine learning approaches? Data were retrieved from one school system available through the proprietary Building Dreams (BD) platform, owned by the Fight for Life Foundation (FFLF), whose mission is to support youth in underserved communities. The BD platform, licensed to K-12 schools, collects student-level data reported by educators on core values associated with in-class participation (a reported—negative or positive—behavior relative to the core values) based on Social–Emotional Learning (SEL) principles. We used a multi-phased approach leveraging several machine learning techniques (clustering, qualitative analysis, classification, and refinement of supervised and unsupervised learning). Unsupervised technique was employed to explore strong boundaries separating students using unlabeled data.

**Results:**

From over 20,000 recorded behaviors, we were able to train a classifier with 90.2% accuracy and uncovered a major underlying factor directly affecting absenteeism: the importance of peer relationships. This is an important finding and provides data-driven support for the fundamental idea that peer relationships are a critical factor affecting absenteeism.

**Discussion:**

The reported results provide a clear evidence that implementing socio-emotional learning components within a curriculum can improve absenteeism by targeting a root cause. Such knowledge can drive impactful policy and programming changes necessary for supporting the youth in communities overwhelmed with adversities.

## Introduction

In a seminal article on attendance differentiation documenting the evolution of the study of absenteeism over the last 100 years, Heyne et al. (2019) leading proponents in the field, shared the etiology and inconsistent presentation of several types of school attendance problems (SAPs; Heyne et al., 2019). The documentation of school refusal (Heyne, 1998), school avoidance, school withdrawal, truancy, and other types and differentiation have continued to inhibit national and international robust studies or evaluations. In fact, finding consistency in outcomes and interventions has also been negatively influenced. This inconsistency has been touted as one of the most challenging dilemmas in defining a clear path forward for attendance intervention (Epstein and Sheldon, 2002); (Heyne et al., 2019). Training educators, counselors, leaders, attendance officers, and other school personnel have been a constant aim (Franklin et al., 2008) as Franklin et al. (2008) pointed out in their school practitioners’ companion to prevent dropouts and attendance problems. Obviously important, the other is in training data for effective outcomes. The training is in the collection and distilling of information and data for use. As such, alongside training and improvement in how to work within schools and respond to attendance problems, collecting and organizing student behavior to inform effective responses has dominated the field in the last 10 years (Ng et al., 2019). Leading scholars, Heyne et al. (2021) on what works and Kearney and Graczyk (2014) on the response to intervention (RTI) model espouse that growth in conceptualizing problematic absenteeism is still fraught with confusion and lack of consensus. In the United States, many states quickly adopted the multi-tiered systems of support (MTSS) approach advanced by Kearney and Graczyk [National Center for Education Statistics (NCES), 2020] but national data on outcomes are still forthcoming. Practitioner and research gaps continue to point to a need to leverage positive behavioral supports to guide behavior analysis (Johnston et al., 2006). MTSS is defined as an approach to response or instruction for which behavioral supports (e.g., Positive Behavioral Interventions and Supports or PBIS) are increasingly offered from intensive to individualized levels (e.g., response to intervention or RTI; IRIS Center, 2019). Kearney and Graczyk (2020) recommend new clusters in using the model to ensure implementation science is applied with the integration of the MTSS model, arguing that

“a multi-tiered system of supports (MTSS) framework emphasizes many aspects that match well with school attendance and its problems, including prevention and a continuum of supports, screening, evidence-based assessment and intervention, problem-solving and data-based decision-making, implementation fidelity, and natural embedding into extant school improvement plans” (p. 316).

The literature spotlights where socio-emotional factors often impede engagement (Epstein and Sheldon, 2002; Rasasingham, 2015). This is because the contributing factors are wide and varied. Researchers have not been able to pinpoint the specific factors (Hocking, 2008) which consistently result in direct changes in engagement. The concern is worldwide, with countries (Mushtaq and Khan, 2012) including Jamaica, also seeking to understand root causes (Cook and Ezenne, 2010).

With this call to explore better and more effective ways to assess and intervene in school attendance problems (SAP)—its legal statutes, school codes, students’ attendance behaviors, and interventions in a school environment, the following research question is proposed: What can we learn about specific underlying factors of absenteeism using machine learning approaches? To fulfill our goal, we conducted research in partnership with Fight for Life Foundation, Butler University, and Indiana University. We leveraged techniques in machine learning to develop an understanding of absenteeism with the mission to provide support to youth in underserved portions of our community. We report herein on a multi-phased approach to use several machine learning techniques to reveal an underlying pattern to absenteeism *via* Social and Emotional Learning (SEL) data collected on the FFLF Building Dreams platform.

The Fight for Life Foundation, founded in 2007, provides schools and counselors additional support for youth to develop the social and emotional qualities to be successful. Explicitly aimed at underserved communities, the foundation’s mission has impacted hundreds of students across 15 different schools in central Indiana. FFLF leverages technology and a unique gamification system with the capability to integrate into a school’s curriculum while simultaneously collecting behavioral data and providing online tools to allow educators and administrators immediate intervention plans and policies. The ability of the system to communicate across applications offers true interoperability. The result is the effortless exchange of data *via* defined data formats, agreed-upon nomenclature, and defined rules for interaction among applications. This relationship brings to light patterns that have the potential to go unnoticed. This data-driven awareness is the basis of the resources FFLF provides to schools to support social–emotional core values and to equip students with the skillsets needed to manage their emotions and relationships. Social and Emotional Learning is the core of FFLF. The fundamental thesis of SEL is that students thrive when their socio-emotional needs are met. We believe that such knowledge can drive impactful policy and programming changes necessary to support the youth in communities overwhelmed with adversity.

## Literature review

Inadequate education and assessment still plague the US with American students scoring lower than many other nations and parents shrugging shoulders in apathy and indifference to education (Bennett et al., 1998; Berliner, 2002; Roesch and Singer, 2013; Buckley et al., 2017). Bennett and colleagues report “A national still at risk” shared then that approximately 20 million high school seniors were unable to do basic math and graduated without knowing the essentials of US history, during a period where over 6 million dropped out of school altogether. As Berliner and Buckely and colleagues continue to affirm standardized testing has its role, but the US is losing its footing. For minority high school students, results were exponentially higher with many leaving without a high school diploma. School and education are essential drivers for a country’s economy. It ensures it has a skilled citizenry to contribute and one not riddled by predictors of antisocial behaviors (Gentle-Genitty, 2010). Therefore, the importance of being in some form of formal education is integral to a country (Balfanz and Byrnes, 2012). Absenteeism is at the heart of these findings (Kearney et al., 2019).

For decades, research has attempted to uncover all risk factors for why students do not persist, work that continues (Gentle-Genitty, 2010; Ngo et al., 2011; Roh and Marshall, 2018; Brouwer-Borghuis et al., 2019). Common aspects are school characteristics (Moscoso, 2000) and maltreatment of bullying (Slade and Wissow, 2007). Some researchers suggest that the impact is in early childhood (Vilsaint et al., 2013), parents and peers are direct drivers on this relationship (Deutsch et al., 2012), and community (Sheldon and Epstein, 2004) yet perceived and observed neighborhood factors and obesity (Ehlers et al., 2005) have been added too. Still, post-traumatic stress and other cognitive impairments play a role (King et al., 2003; Bokhorst et al., 2008). It is likely that even a teacher absence (Finlayson, 2009) and even the categorization of absences influence academic achievement and serve as risk or protective factors (Gottfried, 2009). As socio-ecological approaches spotlight cumulative risk and promotive factors which impact students even those who are non-delinquents (Laan et al., 2010; Roh et al., 2022), lack motivation (Tuan et al., 2005), and are still in early grades (Randle, 1997), we use socio-emotional as a catchall for the many variables which students may present in what impacts absenteeism (Mervilde, 1981; Rothman, 2001).

Large-scale studies involving over 90,000 youth, between kindergarten through the 12 grades, have shown the positive impacts of SEL programs on the improvement of academic performance, reduction of drop-out rates, as well as lower reported cases of drug use and problematic conduct (Durlak et al., 2011; Taylor et al., 2017). The FFLF offers SEL-specific resources to schools to reinforce the criticality of social and emotional aspects within the classroom, especially where poverty is a factor (Ferguson et al., 2007); (Brooks-Gunn and Duncan, 1997). In such communities, the adversities surrounding a student’s daily life require additional support beyond the traditional curriculum (Sun and Shek, 2012). Good social–emotional learning programs do not operate in isolation but help students learn that their decisions determine their consequences while helping them foster skills in coping, self-awareness, and self-control thereby increasing their likelihood of school attendance and successful outcomes.

### Absenteeism

Skedgell and Kearney (2016) reviewed socio-emotional factors and analyzed them in terms of dimensionality (*0–100%*) and categories (*greater internalizing, greater externalizing, and greater family conflict and active-recreational orientation*). Students who were absent for dimensionality for 15–60% of the time from school demonstrated higher presence of internalizing symptoms than those with less or greater absenteeism. The categorical data organized the clusters into:

1. Greater **Internalizing** symptoms
  i. general anxiety, separation anxiety, social phobia, panic, obsessions and compulsions, and depression,
2. Greater **Externalizing** symptoms
  ii. inattention/hyperactivity, rule-breaking behavior, and aggressive behavior, and
3. Greater **family conflict** and lower **active-recreational** orientation (Skedgell and Kearney, 2016).

Simply, dimensionality refers to two factors: (a) the isolation of influence on a studied variable and (b) the determination of incremental impact on a said variable if more or less of the item observed are added. For instance, though we know most students who are absent have some internalizing symptoms, using dimensionality we can learn which students are likely to have internalizing symptoms based on their number of absences. In this case, we know students who were absent 15–60% of the time had greater internalizing symptoms than compared to students with less than 15% of absences and those with higher than 60% of absences. Therefore, if we want to use SEL symptoms to determine when to intervene, based on attendance rates, we must be informed of the thresholds for prevention and intervention to effectively influence attendance behavior.

The findings suggest socio-emotional factors are pivotal to absenteeism; in fact, it is a public health issue for all of us (Kearney, 2008). Because we know truly little about some of the predictive factors like family and community involvement (Sheldon and Epstein, 2004), we continue to see rise in chronic absenteeism, especially for students found to be under-resourced or in poverty (Zhang, 2003); (Reid, 2005). It is essential that we parse through the data collected to ascertain how we can effectively intervene in the understanding of excessive absences and school refusal behavior (Dube and Orpinas, 2009) using models like RTI and MSST to organize (Kearney and Graczyk, 2014) and modern technologies like machine learning (Domingos, 2012).

### Machine learning

Machine learning is defined as the use of task completion through programming of statistical methods, algorithms, and trained or untrained data (Mitchell, 1997). Educators and social scientists are exploring this learning to better serve and respond to their students. Research is growing with the use of machine learning to reveal patterns and predictions in learning students (Gray and Perkins, 2019). In fact, there are studies using fingerprint recognition (Mishra and Trivedi, 2011), face-recognition techniques to track attendance *via* machine learning techniques (Chintalapudi et al., 2018; Rozario and Manjunatha, 2018) and the use of machine learning to assess what influences a student’s perception of a subject being difficult (Suparwito, 2019) or gamification (Toprceanu, 2017).

When exploring other studies where machine learning was used to explore absenteeism, we found a few examining the relationship between asthma and absenteeism (Lary et al., 2019) predictive modeling of student performance (Ng et al., 2021), and attendance autistic students (Jarbou et al., 2022). More is surely available, but these give a glimpse into many types of opportunities for exploration using this method. Yet, as more and more studies emerge, we learn that the model is flexible, but they require good data and time to train. The work we present has taken over 3 years to refine, hypothesize, structure, and train to share the results we present herein. The right models around the right variables are needed to inform what and how we respond to absenteeism using the method. If we train and input only supervised data with little regard to extrapolating unsupervised patterns, then we limit our knowledge for prevention. We will glean only knowledge for what we already know.

Rastrollo-Guerrero et al. (2020) and Albreiki et al. (2021) both conducted comprehensive surveys of recent literature within the space of machine learning applied to data from academic environments. The papers reviewed were chosen from journals with high impact factors and conference proceedings from the most reputable professional conferences, including IEEE and ACM—considered among the “world’s largest technical professional organization dedicated to advancing technology for the benefit of humanity” (IEEE, 2022). The authors reported significant high accuracies from predictive models used in forecasting academic performance; however, 70% of the papers conducted studies at the collegiate level. Furthermore, the authors discussed the high precision of artificial neural networks on behavioral data, as it relates to academic performance, but cited that these approaches constitute a small minority of the researched models, whereas the most common models demonstrating promise were support vector machines (SVM) and naïve Bayes classifiers. Li et al. (2020) present further evidence of the effectiveness of the SVM model when used to predict academic performance; however, the approach is only demonstrated for a target consisting of two classes. These studies demonstrate the effectiveness of machine learning models; however, the evidence is exhibited for university students in the narrow field-of-view of academic performance and drop-out rates. Our proposed work broadens this focus to understand the connection between absenteeism, and other at-risk factors, for elementary school students, while considering the correlation of these factors with social and emotional behaviors. Moreover, we have not found any literature solely focused on the application of machine learning methodologies to the field of Socio-Emotional Learning for understanding absenteeism.

## Theoretical development

Machine learning rests on how we think and organize thought and action. Learning theories inform the methods of machine learning. Cognitivism, in our evaluation, is the most common human behavior theory as it attempts to use observed data to define information retrieval—supervised and unsupervised learning—to organize, store, and learn (Teaching and Learning Cognitivism, 2022) recognizing sometimes cultural biases in instruction (Parrish and Linder-VanBerschot, 2010) drives the extent of the action (Arponen, 2013). Other theories were social cognitive theory and behaviorism (Bandura, 2008; McLeod, 2018). These theories underscore that machines can only share what it has been programmed and must rely also on rational choice, a factor studied in criminology (Akers, 1990) and social work (Gowdy, 1994).

Socio-Emotional Learning SEL refers to an umbrella term for school programs used to support students in developing social and emotional skills and competencies. Their overarching goal is to enhance emotional intelligence and emotional literacy, support social relations, and decrease risks for future academic and social failures (Hoffman, 2009). SEL programs are growing (Elias et al., 2003) and after the pandemic, its growth suggests a national priority (Weissberg and Cascarino, 2013). There is little evidence on SEL’s ability to identify, intervene, or curb specific variables like attendance.

In our proposed methodology, we employed both unsupervised and supervised machine learning models to analyze SEL data. The data were collected from students in kindergarten through sixth grade during the Fall term of the 2021/2022 school year. Supervised models learn the relationship between variables given a known outcome, whereas unsupervised models learn the outcome from inherent patterns. Both techniques are leveraged, first with unsupervised techniques to identify natural groupings. Thereafter, supervised learning methodologies for classifying the remaining data are employed.

The following section summarizes the data collected within the Building Dreams platform, created by the Fight for Life Foundation, and the models trained to identify students at risk for increased absenteeism. We regard risk in terms of dimensionality. If the SEL models can predict or identify the groups of students who may miss or be absent from school prevention and intervention responses may be better deployed. More specifically, the aim of the models is to classify each student into one of three risk classes: red, yellow, and green, representing at-risk, medium-risk, and low-risk students, respectively. With a clear separation between classifications, one can study the factors defining each group to recognize key drivers in behavior and subsequently offer targeted support. For this work, we chose to focus on gaining insight into underlying factors of absenteeism.

### Data collection

The data used in this study were acquired during the Fall 2021 term at a school in central Indiana. This school was selected because of the broad adoption of the Fight for Life Building Dreams platform across all grade levels. Twenty-six thousand seven hundred and forty-one datapoints were collected on 332, K-6, students, where each datapoint characterizes a reported behavior relative to the 10 core values summarized in Table 1. Core values, and the underlying reasons, are reported in either a positive or negative perspective by educators or administrators and are regarded as either in-class participation or related to individual behavior. All reports, positive or negative, are tied to a core value, resulting in an average of 4.2 reports per student per day, with most of all reports originating from teachers. There exists a one-to-many relationship between reported reasons and core values. Engagement with the FFLF program is accomplished through a unique gamification process where students earn or lose yards relative to the game of football. For instance, positive observation of core values is reported as a first down, while negatively recognized behavior is reported as a sack. In serious situations, a sack can result in a student being removed from class and is reported as a red zone. Furthermore, extra points and flags are reported when they demonstrate positive character traits or concerning behavior, respectively. SEL emphasizes the criticality of healthy peer relationships; therefore, core values associated with in-class participation are more heavily weighted since they reflect interactions with others. Extra points and flags are weighted the least but still make an impact on a student’s overall assessment. All educators who participate in the FFLF program undergo a training process for observing and reporting behaviors through the Building Dreams platform.

**Table 1:** Reported core values.

**Core Values**	
**Description**	**Code**
Enthusiastic in class	CV1
Focused within class	CV2
Meet or exceed expectations on assignments	CV3
Demonstrates initiative	CV4
Follow directions	CV5
Respect other’s space	CV6
Respect for physical settings	CV7
Demonstrate accountability	CV8
Respectful communication	CV9
Positive relationships	CV10

The dataset was used to create machine learning models for identifying at-risk, medium-risk, and low-risk students, labeled as red, yellow, and green groups, respectively. The following section summarizes the methodology used for developing a classifier capable of classifying students based on the proportions of reports relative to first downs, sacks, extra points, flags, and red zones. The dataset, $S\in {\mathbb{R}}^{26,741x25}$ is mapped to a new domain, $S^{\prime }\in {\mathbb{R}}^{332x5}$, where each datapoint is defined by a feature vector for each student, $s_{i}$.


$$s_{i}=\left[x_{i}^{fd},x_{i}^{s},x_{i}^{ep},x_{i}^{f},x_{i}^{rz}\right]$$

$$x_{i}^{fp}=\frac{r_{i}^{fp}}{r_{i}},x_{i}^{s}=\frac{r_{i}^{s}}{r_{i}},x_{i}^{ep}=\frac{r_{i}^{ep}}{r_{i}},x_{i}^{f}=\frac{r_{i}^{f}}{r_{i}},x_{i}^{rz}=\frac{r_{i}^{rz}}{r_{i}}$$

(1) $$r_{i}=r_{i}^{fp}+r_{i}^{s}+r_{i}^{ep}+r_{i}^{f}+r_{i}^{rz}$$

Where $r_{i}^{fp},r_{i}^{s},r_{i}^{ep},r_{i}^{f},and\;r_{i}^{rz}$, denote the total reports of first downs, sacks, extra points, and red zones, respectively, for student $s_{i}$.

## Methodology

**Figure 1:**
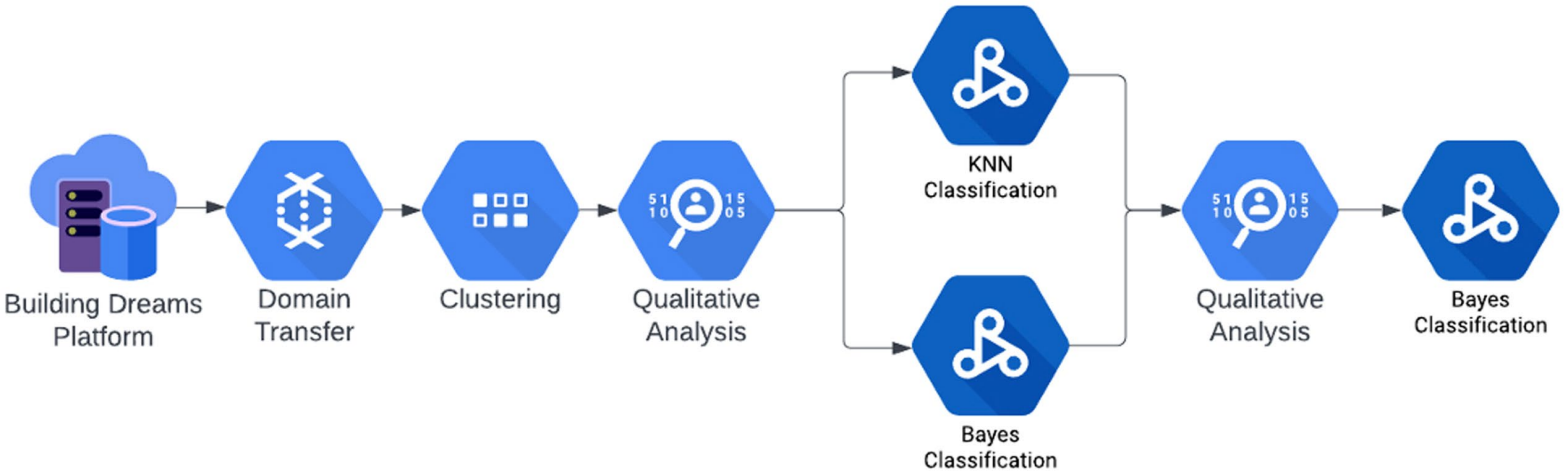
Overview of proposed methodology.

The proposed methodology is a coupling of unsupervised and supervised models, leading to a model for classifying students as at risk, medium risk, and low risk. Data reported by educators per student are unlabeled; therefore, an unsupervised technique is employed to explore strong boundaries separating students. Figure 1 illustrates the entire proposed methodology for developing an effective machine learning model for the classification of behavior data from the Building Dreams platform.

### Clustering and initial label qualitative analysis

In this work, K-means clustering, and qualitative analysis, was leveraged at a classroom level for identifying three classes of students, $C=\left\{C_{LR},C_{MR},C_{HR}\right\}$, characterizing low-risk, medium-risk, and high-risk students, respectively. With this unsupervised model, no prior assumptions about outcome are made and are often used as an exploratory step in many machine learning methodologies. Three classes of risk were chosen to highlight two extremes, high vs. low risk, and identify remaining datapoints. The aim of any clustering model is to find natural groupings of data, called clusters, where each datapoint within a cluster is highly similar, yet datapoints between clusters are highly dissimilar.

Datapoints from each classroom are independently clustered into three clusters where K-means clustering aims to create K clusters by minimizing within-cluster distance. In this work, the Euclidean distance was used as the cost function to minimize. For a set of students in a classroom, $\Gamma_{i}=\left\{s_{1},s_{2},..s_{n}\right\}\subseteq S^{\prime }$, and set of three clusters,$G=\left\{G_{1},G_{2},G_{3}\right\}$, the iterative clustering algorithm is defined by the optimization problem,


$$\underset{G}{\min}\underset{G_{i}}{\sum }\underset{\Gamma_{i}\in G}{\sum }\Gamma_{i}-c_{G_{i}}{}^{2}$$

(2) $$c_{G_{i}}=\left[\bar{x_{i}^{fd}},\bar{x_{i}^{s}},\bar{x_{i}^{ep}},\bar{x_{i}^{f}},\bar{x_{i}^{rz}}\right],\forall s_{j}\in G_{i},$$

The cluster centers, $c_{G_{i}}$, are evaluated qualitatively to map $G_{i}\to C_{j}$, and all classroom-level clusters are assigned labels, $C_{LR},C_{MR},or\;C_{HR}$. The process is repeated for all 15 classrooms, resulting in 45 feature vectors associated with the desired class labels. Of the 15 classrooms, nine clusters made sense from the qualitative analysis, with clear separation between the clusters. The resulting 27 cluster centers from those nine classrooms were used as training data for two classifier models used to predict the class label for the remaining six classrooms.

### Classification

Classification models are trained in a supervised manner where a set of features are associated with a known class label. In this work, we examined the results of the clustering model that are then used to train a classifier model for associating a risk label to a student’s feature vector, comprised of their percent reports from each of the different report types of first downs, sacks, extra points, flags, and red zones. Each cluster is characterized by a vector defined in (2), and is associated with a risk label assigned in the previous phase.

After initial labels are determined, two classification models are trained on the cluster centers $c_{G_{i}}$that were successfully labeled in the previous phase. Naïve Bayes classifiers rely on the conditional probability that a given feature vector, $s_{i}$, belongs to $C_{j}$.


(3) $$p\left(C_{j}\;|x_{i}^{fd},x_{i}^{s},x_{i}^{ep},x_{i}^{f},x_{i}^{rz}|\right)$$

Since $\left\{s_{i}\in \mathbb{R}|0\leq s_{i}\leq 1\right\}$, the Gaussian Naïve Bayes classifier is used to estimate the likelihood component of Bayes theorem, highlighted in (4), relying on a Gaussian distribution defined from the mean and standard deviations of each feature in the training sets.


(4) $$\begin{array}{l} p\left(C_{j}\;|\;x_{i}^{fd},x_{i}^{s},x_{i}^{ep},x_{i}^{f},x_{i}^{rz}\right)\\ =\frac{p\left(C_{j}\right)\;p\left(x_{i}^{fd},x_{i}^{s},x_{i}^{ep},x_{i}^{f},x_{i}^{rz}\;,\;C_{j}\right)}{p\left(x_{i}^{fd},\;x_{i}^{s},\;x_{i}^{ep},\;x_{i}^{f},\;x_{i}^{rz}\right)} \end{array}$$

Bayes classifiers operate on conditional probabilities defined by an entire training set, whereas K-nearest neighbor (KNN) classifiers assign class labels based on feature similarity within an evaluation set. A class label is defined by the most common label residing within the evaluation set of the K most similar datapoints. In this work, the Euclidean distance (5) was used as the similarity measure driving the decision process of the KNN classifier.


(5) $$d\left(s_{i},\;s_{j}\right)=\sqrt{\begin{array}{l} {\left(x_{i}^{fd}-x_{j}^{fd}\right)}^{2}+{\left(x_{i}^{s}-x_{j}^{s}\right)}^{2}+{\left(x_{i}^{ep}-x_{j}^{ep}\right)}^{2}\\ +{\left(x_{i}^{f}-x_{j}^{f}\right)}^{2}+{\left(x_{i}^{rz}-x_{j}^{rz}\right)}^{2} \end{array}}$$

The ideal neighborhood size, K, was found empirically by training and evaluating models over the entire viable range. For this work, a neighborhood size of five was found to produce the most accurate classifier for the available data.

### Label refinement, qualitative analysis, and final classifier model

Both classifiers are trained on the high confidence data from the previous phase and then used to predict the class labels on the data with less confidence after the initial clustering and qualitative analysis. The resulting prediction from each classifier is compared where a label is assumed to be accurate when both classifiers agree in the outcome; however, when the two classifiers produced different predictions, a qualitative analysis of the data is performed to manually decide the appropriate label or decide if the cluster should be completely disregarded. The final cluster centers from all classrooms then become the training set for a generalized Bayes classifier used to label all current and future students.

## Results and discussion

**Figure 2:**
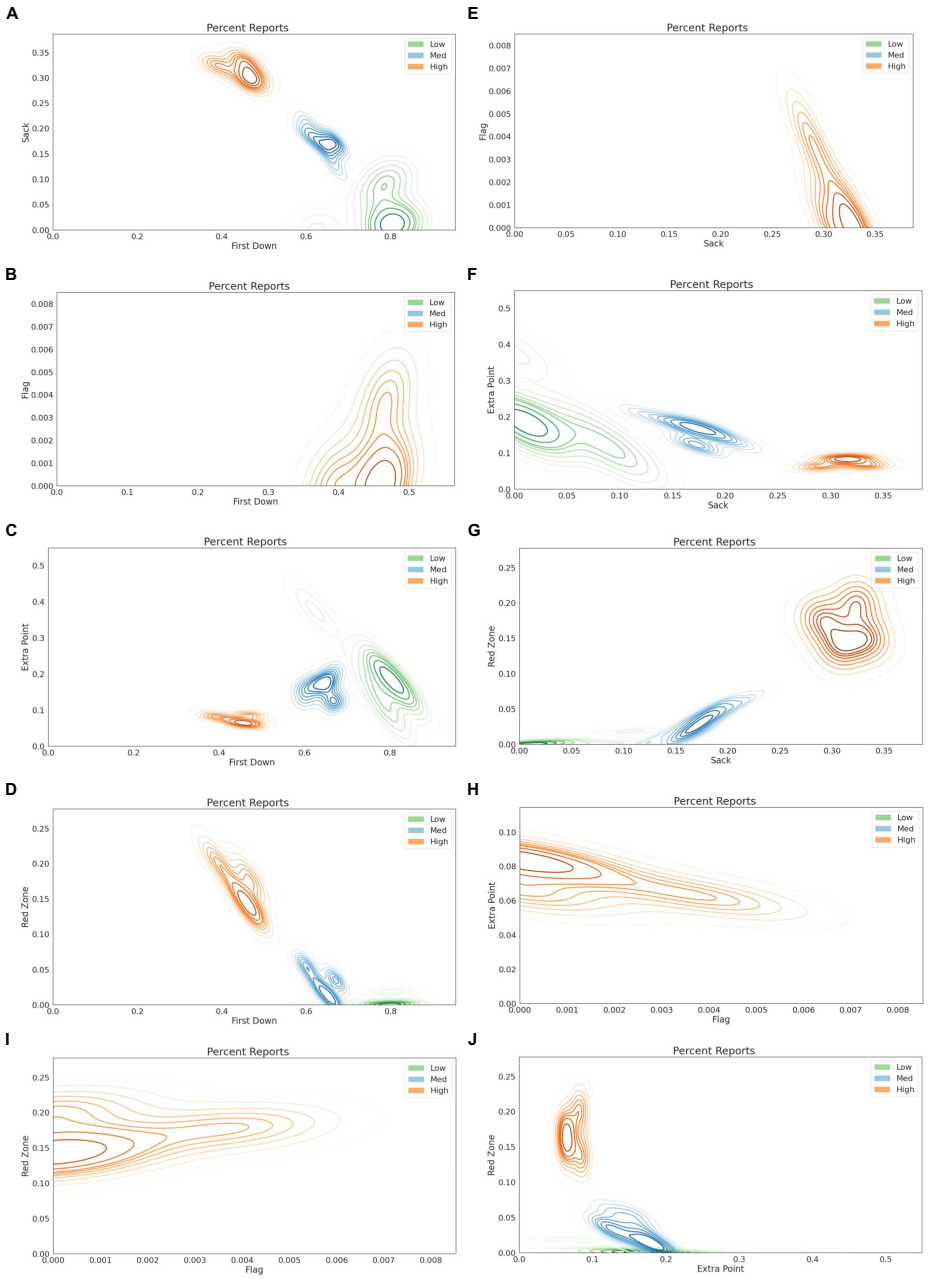
Sample clustering visualizations for a single classroom, showing distributions of percent reports for first down vs. sacks **(A)**, first down vs. flags **(B)**, first down vs. extra points **(C)**, first down vs. red zones **(D)**, sacks vs. flags **(E)**, sack vs. extra points **(F)**, sacks vs. red zones **(G)**, flags vs. extra points **(H)**, flags vs. flags **(I)**, and extra points vs. red zones **(J)**.

**Figure 3:**
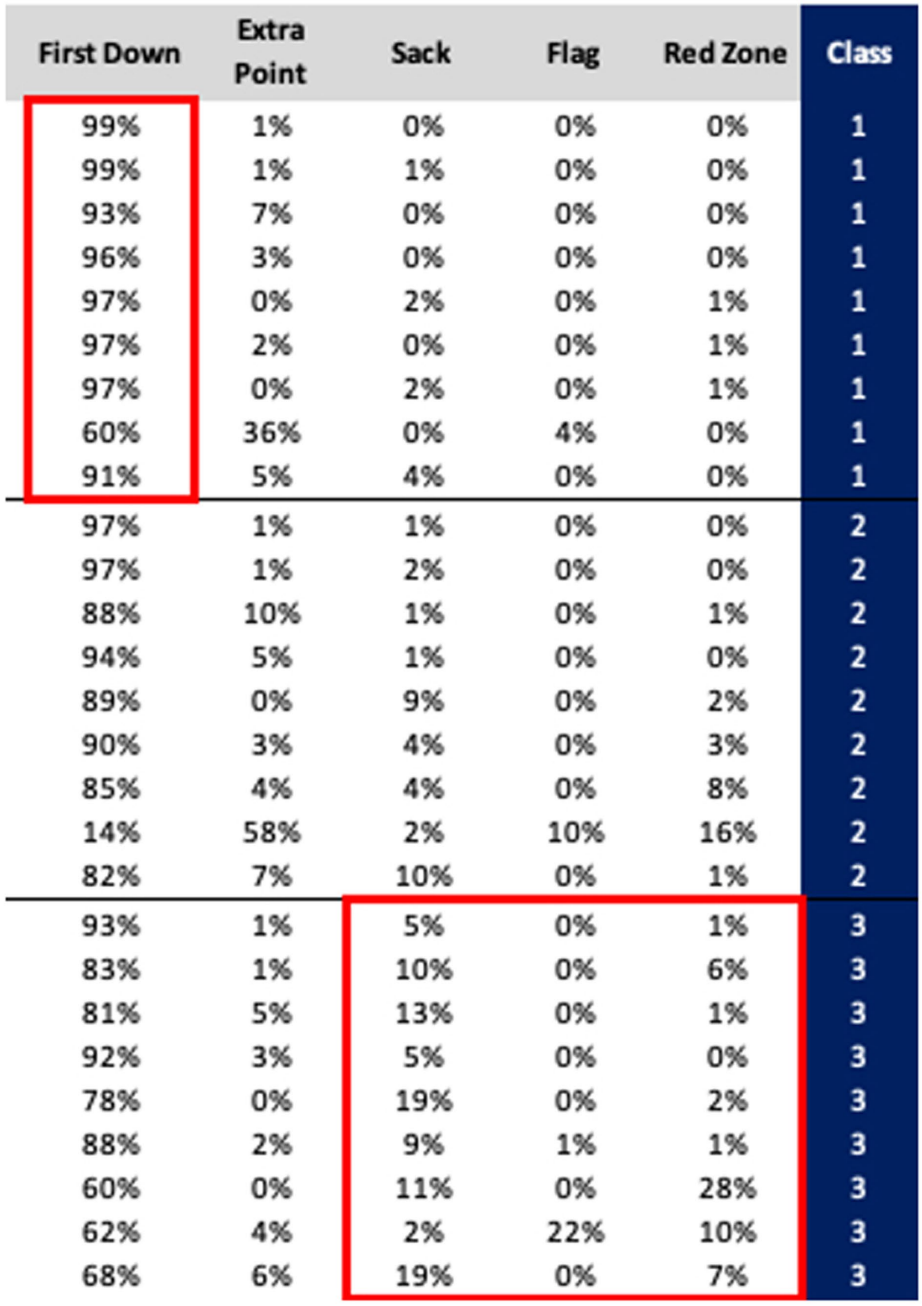
Average cluster centers per classroom.

Figure 2 provides a visualization of the clustering results for a single classroom, illustrating the most critical features that differentiate the clusters, while Figure 3 summarizes the cluster centers for nine of the 15 classrooms. For the example shown, first downs, sacks, and red zones, appear to be strong differentiators of the clusters. This pattern is also observed in Figure 3, where $C_{LR}$ is defined by values first downs and lower percent reports of sacks and red zones. Conversely, $C_{HR}$, is characterized by the lowest percent reports of first downs and highest occurrences of sacks and red zones. Visualizations for all classrooms were generated and evaluated to associate each classroom-level cluster with the most appropriate label, $C_{i}$. Clustering was performed on all classrooms, resulting in 45 datapoints from the three clusters for each of the 15 classrooms; however, nine of the 15 classrooms naturally fit into highly differentiated clusters. The highlighted features in Figure 3 were used to determine that clusters 1–3, exemplify low-risk, medium-risk, and high-risk students, respectively. We have found, and presented visually, a clear separation between low- and high-risk clusters.

**Figure 4:**
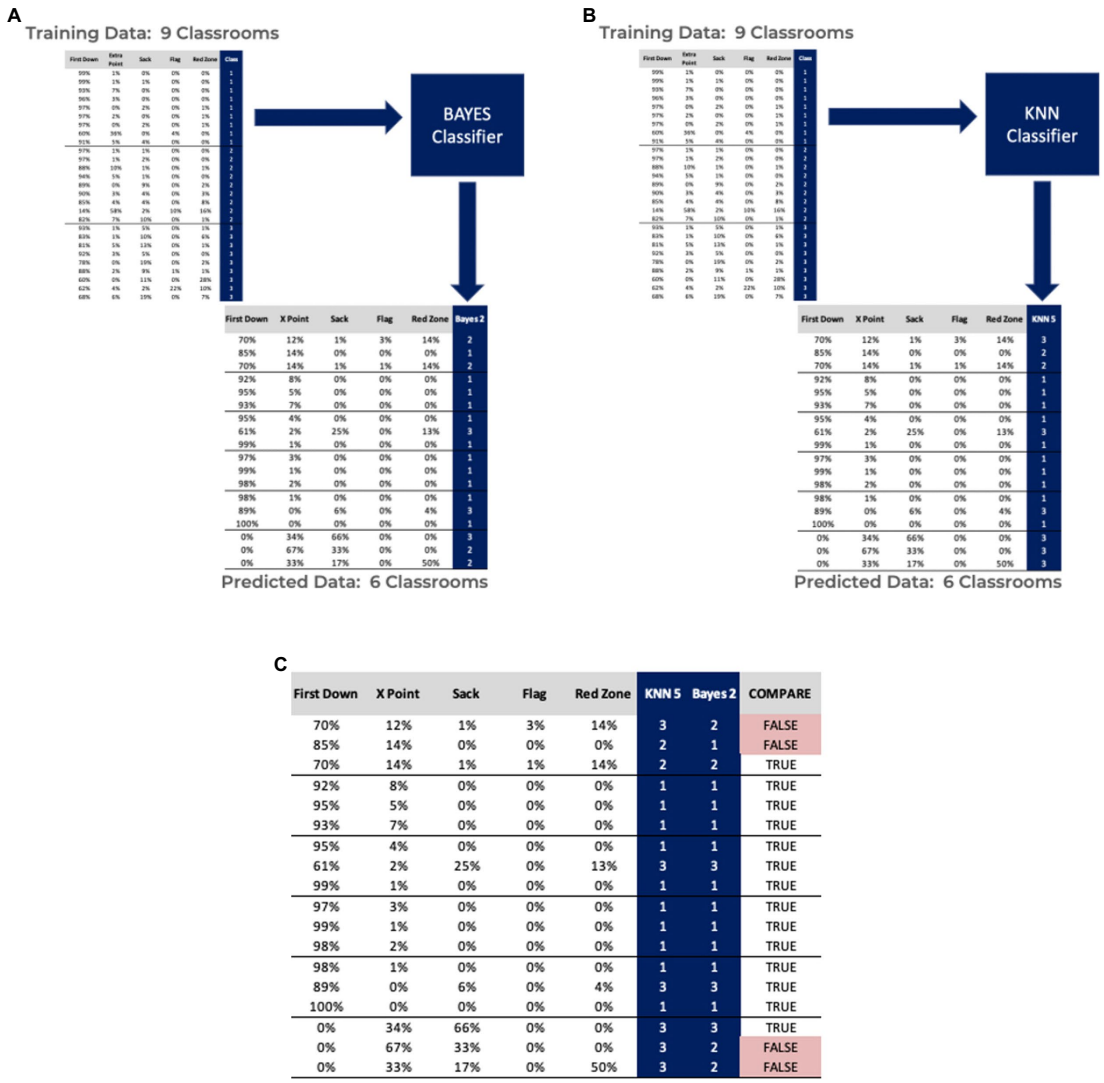
Results of using a Bayes **(A)** and KNN **(B)** classifiers, trained on labeled data from the clustering phase. Comparing classifier results when applied to the clustered data that was not easily differentiated **(C)**.

Figure 4, as well as Tables 2, 3 summarize the results from the classification phase of the proposed methodology. The confusion matrices for Bayes and KNN classification steps demonstrate accuracies of 77.8 and 63.0%, respectively. After a second round of qualitative analysis is performed, accepting all labels where the two classifiers agreed, a total of four entries are rejected as outliers and discarded. Further analysis of this classroom data reveals inconsistent reporting behavior from the educators. For instance, as observed in Figure 4C, one classroom did not report any first downs and simply used the Building Dreams platform for recognizing two of the five categories. After completion of the second round of data classification, it is apparent that some classrooms simply do not have three classifications of students, which is the primary disadvantage of the first step where the K-means algorithm attempts to create three distinct groups. We believe we have overcome this drawback by only accepting the clustering results that were observed to be obvious and then training classifiers to attempt to label the remaining data.

**Table 2:** Confusion matrix for Bayes Classifier.

	*C*_*LR*_	*C*_*MR*_	*C*_*HR*_
*C*_*LR*_	8	0	1
*C*_*MR*_	2	5	2
*C*_*HR*_	0	1	8

**Table 3:** Confusion matrix for KNN Classifier.

	*C*_*LR*_	*C*_*MR*_	*C*_*HR*_
*C*_*LR*_	7	1	1
*C*_*MR*_	1	6	4
*C*_*HR*_	1	2	6

The resulting 41 cluster centers and associated labels were used to train a final Bayes classifier that was evaluated to be 90.2% accurate. This classifier, trained at the classroom level, was applied to student data from the end of the Fall 2021 term. Table 4 summarizes the number of students, in addition to the average feature for each class after employing the final model. In the subsequent section, we investigated how this classifier can be used to better understand underlying factors affecting absenteeism.

**Table 4:** Average features per class after applying final classifier to students in a validation set.

Class	Student count	First down	Extra points	Sacks	Flags	Red zones
*C*_*LR*_	210	96%	4%	1%	0%	0%
*C*_*MR*_	68	89%	3%	7%	0%	1%
*C*_*HR*_	54	63%	9%	21%	1%	6%

### Application of the final classification model

The proposed methodology for training an effective classifier was pursued with the purpose of better understanding the needs of at-risk students. There are many areas that could benefit from understanding the difference between low- and high-risk students. We specifically focused on absenteeism, a major issue affecting youth in underserved communities. In this subsection, we will discuss the trends in the data after applying the classification model for identifying low-, medium-, and high-risk students. The goal was to uncover insights by comparing trends from data labeled as $C_{LR}$ vs. $C_{HR}$. The labels generated by the trained classifier were applied to the original dataset then descriptive analytics was leveraged to analyze the original reported reasons and associated core values. The following observations were made while comparing distributions of reported core values, and their underlying reasons, of students in the $C_{LR}$ and $C_{HR}$ groups with the intention of understanding what differentiates each group and gain insights into commonalities that are actionable.

The first observation is the noticeable discrepancy of reported data directly tied to attendance. Comparing $C_{LR}$ and $C_{HR}$ groups, 99.6% of positive reports of a student attending class on time are labeled with $C_{LR}$. Similarly, for the positively observed behavior of “reporting to class prepared to learn,” 91.4% of the reports is associated the $C_{LR}$ group, but only 8.6% of the reports is associated with the $C_{HR}$-labeled students. In terms of overall reports, across the entire dataset for all three groups, students attending class on time account for 6.3% of the positive reports for $C_{LR}$ students, whereas only 1.7% in the $C_{HR}$ group. Students in the $C_{LR}$ group are notably characterized by the top three reports of following directions (9.8%), contributing to class discussions (7.74%), and reporting to class on time (6.3%), whereas the $C_{HR}$ group is recognized for those same reasons infrequently, accounting for only 3.34, 3.1, and 1.7%, respectively, of total positive reports. The top three reported reasons in the $C_{HR}$ group are negative observations for not following directions (10.3%), not follow rules (4.5%), and fighting (3.26%), where the same observations in the $C_{LR}$ group only accounts for 0.36%, 0.02%, and 0.01% of the total reports. We see from these distributions, by comparing reported reasons across $C_{LR}$ and $C_{HR}$ groups, as well as looking at reported reasons over the entire dataset, absenteeism is a differentiating factor for students labeled by the classifier as low or high risk.

**Figure 5:**
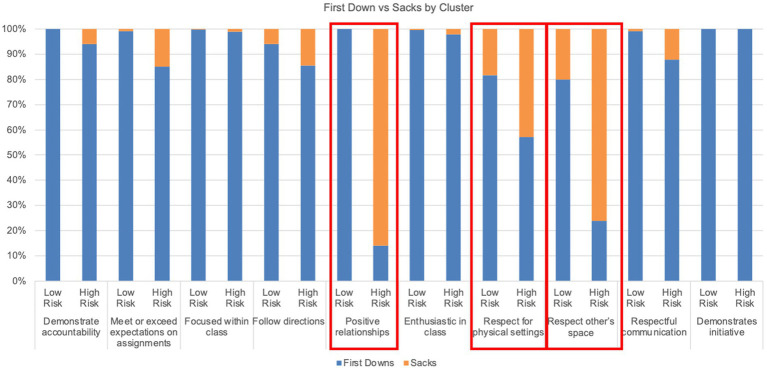
Core value comparison between *C*_*LR*_ and *C*_*HR*_ labeled data.

**Figure 6:**
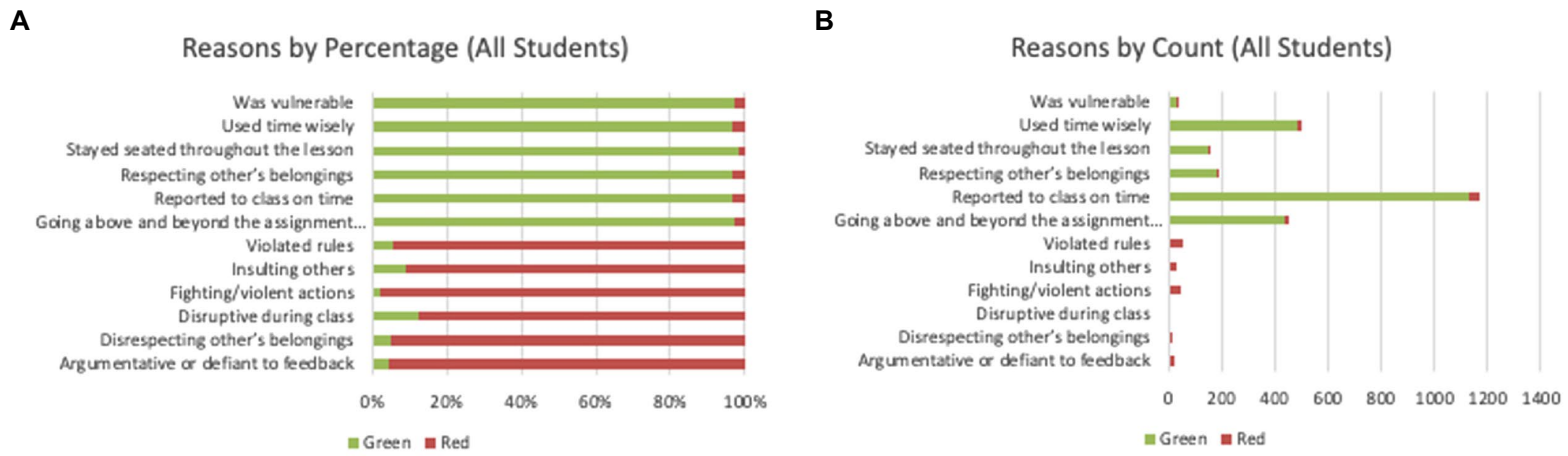
Reported reasons comparison between *S*_*LR*_ (green) and *C*_*HR*_ (red) labeled data.

Each reported reason is associated with one of the 10 core values reported in Table 1. A similar exercise was conducted to compare the labeled dataset but in terms of core values instead of reported reasons. In Figure 5, we see that the top three differences between high-risk and low-risk student groups are the core values related to peer relationships. Furthermore, we looked at the underlying reasons reported along with the core values. Figure 6 summarizes the most common differences between $C_{LR}$ and $C_{HR}$ data, in terms of underlying reasons. Four of the six reported reasons for the high-risk group are related to peer relationships. Conversely, it is immediately apparent that the low-risk group’s most reported reasons are a positive recognition of attendance, while the high-risk group is rarely recognized for the same behavior.

**Figure 7:**
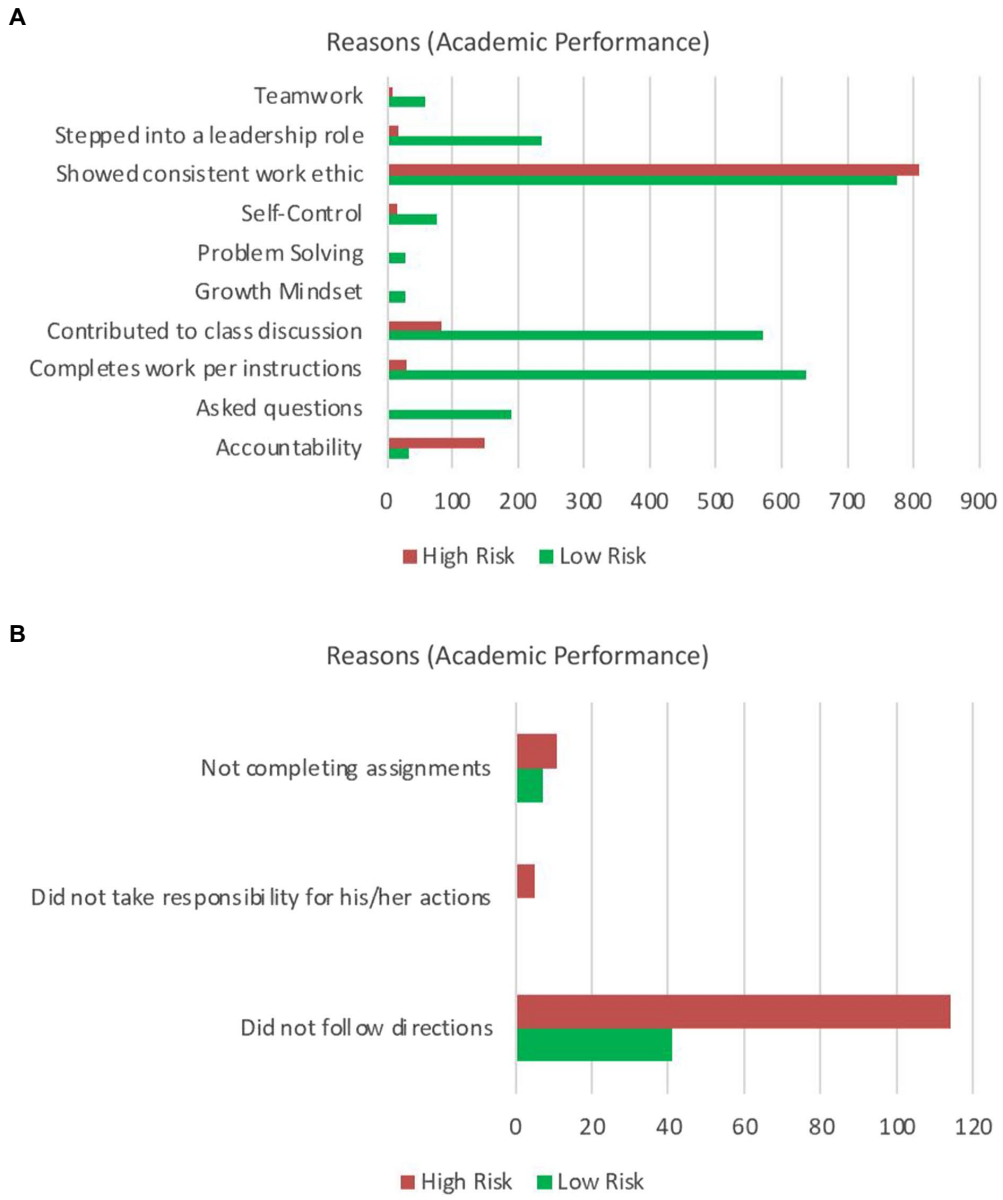
Reason related to academic performance that could be reported as either being positively or negatively observed as either a first down or sack, respectively **(A)**, and reported reasons related to academic performance that are only negatively observed as sacks or red zones **(B)**.

Academic performance was observed to be another key differentiator of high- and low-risk students. When analyzing reasons reported by the teachers, we noticed that there was a clear disparity in reasons directly tied to academic performance. These reasons are summarized in Figure 7, where one subset of reasons could be recognized in either a positive or negative perspective, while another subset of reasons could only be interpreted as a negative report, in Figures 7A,B, respectively. The examples in Figure 7A illustrate how the low-risk group was reported for the same reasons as the high-risk group, but in a positive context instead of a negative one, while Figure 7B provides example reasons that were only cited in a negative context and show a large discrepancy between opposing risk groups. The low-risk student groups were cited for having strong work ethics, contributing to class discussion, and completed course work per the instructions, while only being cited for not following directions 60% less than the high-risk groups. Conversely, the high-risk students were found to be cited for showing consistent work ethic, but in a negative perspective, as often as low-risk students are recognized in a positive way for the same reason. Both charts illustrate that the high-risk students are responsible for many of the reports related to accountability but were always negatively observed.

### Discussion

Even though the importance of understanding absenteeism and its impact on students’ (Skedgell and Kearney, 2016) and even entire countries’ economies have been widely studied (Cook and Ezenne, 2010; Mushtaq and Khan, 2012; IRIS Center, 2019; Kearney and Graczyk, 2020), there is a lack of consensus in specific factors contributing to absenteeism as well as coordinated assess and interventions (Sheldon and Epstein, 2004; Hocking, 2008). Our work sheds light on this important issue by identifying specific underlying factors in students’ behaviors connected to absenteeism. Our data-driven approach indicated with 90% accuracy that peer relationships are at the core of absenteeism underlying factors. These are relevant findings because the data supports the key idea that peer relationships are a critical factor affecting absenteeism and provides clear evidence that the implementation of socio-emotional learning components within a curriculum has the potential to improve absenteeism by targeting a root cause. The clear discrepancy between low- and high-risk students for reporting to class on time, and reporting to class prepared, exemplifies how each group of students differ in terms of attendance. Recognizing this difference but considering other differentiating factors, we observe that academic performance and peer relationships also distinctly separate the two groups. Academic performance differences are easily recognized through reported reasons while disparities in peer relationship reports are evident under the lens of core values, as illustrated in Figure 5. These are relevant findings because the data support the key idea that peer relationships are a critical factor affecting absenteeism.

We argue that although absenteeism has many driving factors, such as external socio-economic factors, we can narrow the focus to a specific set of problems from primary data collected on-site, such as peer relationships. Although not the only factor of absenteeism, peer relationships were brought into focus using the proposed machine learning methodologies. The reported reasons associated with peer relationship-based core values, such as being argumentative, fighting, disrespecting other’s belongings, insulting one’s peers, and threatening others, are commonly reported behaviors for students in the high-risk group, but rarely, if at all, are observed in the low-risk groups. These reports give a targeted direction for new SEL curriculum and school policy. The proposed methodology for classifying students was used as a tool to support the belief that absenteeism is also highly correlated with poor academic performance. Through the process of labeling the data with the classification system, we were able to find observed behavior, directly related to academic performance, for comparing at-risk and low-risk students. Work ethic and the ability of a student to follow directions are the two reported reasons directly affecting academic performance that helps define high- and low-risk student groups. Each of those reported reasons is reported heavily for both groups, but in opposite perspectives; first downs versus sacks. Furthermore, the low-risk students were found to ask questions, while no students in the high-risk group were ever cited for the same observation.

There are various studies on predicting attendance and relationships with academic performance. Many are based on data that researchers and educators have long held as hypotheses that have been proven. However, the current study aimed to get at whether we see those same outcomes from unsupervised data and patterns. We are happy to confirm that we do. It is affirming to see that relationships consistently are supported as predictors, thus supporting the role of SEL programs as the most effective at reducing absenteeism.

Limitations of machine learning techniques can be viewed in terms of the models used, data, and process. With unsupervised learning models, such as clustering algorithms, an outcome is unknown and often requires human intervention to interpret the results. As such, clustering algorithms are frequently used as an exploratory tool. Moreover, the decision process during clustering requires a metric for measuring similarity, with the Euclidean distance being the most common method; however, the choice of a similarity metric can affect the overall results. In this work, the separation of the clusters was analyzed visually (Figure 2) and each cluster was analyzed statistically (Table 4) to rationalize the effectiveness of the results while using the Euclidean distance for identifying the appropriate clusters. In supervised learning algorithms, such as regression or classification, a known outcome is related to the variables. The choice of model can also influence the overall results. For instance, the K-Nearest Neighbor classifier relates the data label to the variables through a similarity metric, while the Bayes classifier predicts a label based on a conditional probability and the application of Bayes Theorem. Depending on the training set, both classifiers can offer different perspectives on a predicted label. In the proposed system, we combine both perspectives to identify risk labels. Other classifiers, including support vector machines, random forest classifiers, and neural networks, offer alternative approaches to accomplish the same task. Regardless of the model selection, the data are the most impactful limiting factor for machine learning. One must strive for large amounts of high-quality data, where high-quality broadly refers to data that accurately represent the population and are consistent over time. These two requirements rely on the proper processes and technology for data collection and curation. The Building Dreams platform is built on several years of development and deployed with a rigorous training program to achieve the accuracy and consistency needed to confidently train machine learning models.

## Future work

In this work, we focused on a single term while looking at a specific area affecting the youth of a single school. Future work includes answering additional research questions about academic performance and drop-out rates, while applying and validating the models to additional data from future terms and other schools. We recognize that this student applies a classification model to produce data labels using primary data collected at the school. Future work will correlate external factors into the models. Lastly, additional classification models, such as support vector machines, random forest classifiers, and neural networks, may be explored and compared to the proposed methodologies.

## Conclusion

The work presented in this paper signifies the initial steps taken to leverage machine learning techniques on SEL data to better understand the areas that could make a relevant impact in the lives of children in underserved communities. In collaboration with the Fight for Life Foundation, we have developed a classification model that was used to examine absenteeism. The proposed multi-phased approach was evaluated to be 90.2% accurate in identifying three classes of students: low risk, medium risk, and high risk. Future work will focus on looking at other factors differentiating these groups, such as academic performance and drop-out rates with the ultimate mission of providing support in an effective and targeted manner.

## Data availability statement

The original contributions presented in the study are included in the article/supplementary material, further inquiries can be directed to the corresponding author.

## Author contributions

FB, CG-G, JS, and MJ contributed equally to the data, assessment, and production of the work submitted. All authors contributed to the article and approved the submitted version.

## Funding

This work and its exploration were funded by Butler University and Indiana University (IUPUI) Chancellor Bantz Community Scholar Fellowship.

## Conflict of interest

The authors declare that the research was conducted in the absence of any commercial or financial relationships that could be construed as a potential conflict of interest.

## Publisher’s note

All claims expressed in this article are solely those of the authors and do not necessarily represent those of their affiliated organizations, or those of the publisher, the editors and the reviewers. Any product that may be evaluated in this article, or claim that may be made by its manufacturer, is not guaranteed or endorsed by the publisher.

## References

[ref1] Akers R. L. (1990). Rational choice, deterrence, and social learning theory in criminology: the path not taken. J. Crim. Law Criminol. 81:653. doi: 10.2307/1143850

[ref01] Albreiki B. Zaki N. Alashwal H. (2021). A systematic literature review of student’ performance prediction using machine learning techniques. Education Sciences 11:552.

[ref2] Arponen V. P. (2013). The extent of cognitivism. Hist. Hum. Sci. 26, 3–21. doi: 10.1177/0952695113500778

[ref3] Balfanz R. Byrnes V. (2012). The importance of being in school: A report on absenteeism in the Nation's public schools. Available at: http://new.every1graduates.org/wp-content/uploads/2012/05/finalchronicabsenteeismreport_may16.pdf (Accessed April 3, 2022).

[ref4] Bandura A. (2008). Social cognitive theory. Available at: https://uky.edu/~eushe2/bandura/bandura1989acd.pdf (Accessed April 3, 2022).

[ref5] Bennett W. J. Fair W. Finn C. E. Flake F. H. Hirsch E. D. Marshall W.. (1998). A nation still at risk. Pol. Rev. 90, 23–29.

[ref6] Berliner B. J. (2002). Educational leadership:Beyond instructional leadership:Unequal school funding in the United States. Available at: http://www.ascd.org/publications/educational-leadership/may02/vol59/num08/Unequal-School-Funding-in-the-United-States.aspx (Accessed October 15, 2022).

[ref7] Bokhorst C. L. Westenberg P. M. Oosterlaan J. Heyne D. (2008). Changes in social fears across childhood and adolescence: age-related differences in the factor structure of the fear survey schedule for children-revised. J. Anxiety Disord. 22, 135–142. doi: 10.1016/j.janxdis.2007.01.014, PMID: 17339097

[ref8] Brooks-Gunn J. Duncan G. J. (1997). The effects of poverty on children. Futur. Child. 7, 55–71. doi: 10.2307/1602387, PMID: 9299837

[ref9] Brouwer-Borghuis M. L. Heyne D. Vogelaar B. Sauter F. M. Scholte R. H. (2019). The link: an alternative educational program in the Netherlands to reengage school-refusing adolescents with schooling Cognitive and Behavioral Practice 26, 75–91. doi: 10.1016/j.cbpra.2018.08.001

[ref10] Buckley J. Letukas L. Wildavsky B. (2017). Measuring Success: Testing, Grades, and the Future of College Admissions, Johns Hopkins University Press.

[ref11] Chintalapudi S. K. Reddy P. Tatapudi K. S. (2018). Attendance using face recognition. Int. J. Adv. Res. Comput. Sci. 9, 49–51. doi: 10.26483/ijarcs.v9i6.6339, PMID: 36320405

[ref12] Cook L. D. Ezenne A. (2010). Factors influencing Students' absenteeism in primary schools in Jamaica: perspectives of community members. Carib. Curric. 17, 33–57.

[ref13] Deutsch A. R. Crockett L. J. Wolff J. M. Russell S. T. (2012). Parent and peer pathways to adolescent delinquency: variations by ethnicity and neighborhood context. J. Youth Adolesc. 41, 1078–1094. doi: 10.1007/s10964-012-9754-y, PMID: 22460729

[ref14] Domingos P. (2012). A few useful things to know about machine learning. Commun. ACM 55, 78–87. doi: 10.1145/2347736.2347755

[ref15] Dube S. R. Orpinas P. (2009). Understanding excessive school absenteeism as school refusal behavior. Child. Sch. 31, 87–95. doi: 10.1093/cs/31.2.87

[ref06] Durlak J. A. Weissberg R. P. Dymnicki A. B. Taylor R. D. Schellinger K. B. (2011). The impact of enhancing students’ social and emotional learning: a meta-analysis of school-based universal interventions. Child Development 82, 405–432. 21291449 10.1111/j.1467-8624.2010.01564.x

[ref16] Ehlers S. Lehmann J. Mossmann H. Alber G. Hölscher C. (2005). Interleukin-12p40 mediates transient protection against Mycobacterium avium infection in the absence of interleukin-12. Immunobiology 210, 217–227. doi: 10.1016/j.imbio.2005.05.016 16164029

[ref08] Elias M. J. Zins J. E. Graczyk P. A. Weissberg R. P. (2003). Implementation, sustainability, and scaling up of social-emotional and academic innovations in public schools. School Psychology Review 32, 303–319.

[ref17] Epstein J. Sheldon S. (2002). Present and accounted for: improving student attendance through family and community involvement. J. Educ. Res. 95, 308–318. doi: 10.1080/00220670209596604

[ref18] Ferguson H. Bovaird S. Mueller M. (2007). The impact of poverty on educational outcomes for children. Paediatr. Child Health 12, 701–706. doi: 10.1093/pch/12.8.701, PMID: 19030450 PMC2528798

[ref19] Finlayson M. (2009). The impact of teacher absenteeism on student performance: The case of the Cobb County School District. Available at: https://digitalcommons.kennesaw.edu/etd/4 (Accessed April 3, 2022).

[ref20] Franklin C. Harris M. Allen-Meares P. (2008). The School Practitioner's Concise Companion to Preventing Dropout and Attendance Problems, Oxford University Press.

[ref21] Gentle-Genitty C. (2010). Common predictors for explaining youth antisocial behavior: a perspective from ten longitudinal studies. Soc. Work. Ment. Health 8, 543–559. doi: 10.1080/15332980902983824

[ref22] Gottfried M. A. (2009). Excused versus unexcused: how student absences in elementary school affect academic achievement. Educ. Eval. Policy Anal. 31, 392–415. doi: 10.3102/0162373709342467

[ref23] Gowdy E. A. (1994). From technical rationality to participating consciousness. Soc. Work 39, 362–370.

[ref24] Gray C. C. Perkins D. (2019). Utilizing early engagement and machine learning to predict student outcomes. Comput. Educ. 131, 22–32. doi: 10.1016/j.compedu.2018.12.006, PMID: 35777803

[ref25] Heyne D. (1998). Introduction to the special series on school refusal. Behav. Chang. 15, 3–4. doi: 10.1017/S0813483900005854

[ref02] Heyne D. Brouwer-Borghuis M. Vermue J. Van Helvoirt C. Aerts G. J. W. (2021). Knowing what works: A roadmap for school refusal interventions based on the views of stakeholders. NRO Nationaal Regieorgaan Onderwijsonderzoek. Netherlands Initiative for Education Research. Avialable at: https://www.nro.nl/sites/nro/files/media-files/Eindrapport%20Knowing%20what%20works%20%28nov.%202021%29.pdf.

[ref26] Heyne D. Gren-Landell M. Melvin G. Gentle-Genitty C. (2019). Differentiation between school attendance problems: why and how? Cogn. Behav. Pract. 26, 8–34. doi: 10.1016/j.cbpra.2018.03.006

[ref27] Hocking C. (2008). The contributing factors to student absenteeism/truancy and the effectiveness of social services and interventions. Available at: http://digitalcommons.providence.edu/cgi/viewcontent.cgi?article=1017&context=socialwrk_students (Accessed April 3, 2022).

[ref03] Hoffman D. M. (2009). Reflecting on social emotional learning: a critical perspective on trends in the United States. Review of Educational Research 79, 533–556.

[ref04] IEEE (2022). IEEE (Institute of Electrical and Electronics Engineers). Avilable at: https://www.ieee.org/about/index.html?utm_source=dhtml_footer&utm_medium=hp&utm_campaign=learn-more

[ref28] IRIS Center (2019). Definition: Multi-tiered system of support. Nashville: IRIS

[ref29] Jarbou M. Won D. Gillis-Mattson J. Romanczyk R. (2022). Deep learning-based school attendance prediction for autistic students. Sci. Rep. 12, 1–11. doi: 10.1038/s41598-022-05258-z 35082310 PMC8791997

[ref30] Johnston J. M. Foxx R. M. Jacobson J. W. Jacobson J. W. Green G. Green G.. (2006). Positive behavior support and applied behavior analysis. Behav. Anal. 29, 51–74. doi: 10.1007/BF03392117, PMID: 22478452 PMC2223172

[ref31] Kearney C. A. (2008). School absenteeism and school refusal behavior in youth: a contemporary review. Clin. Psychol. Rev. 28, 451–471. doi: 10.1016/j.cpr.2007.07.012, PMID: 17720288

[ref32] Kearney C. A. Gonzálvez C. Graczyk P. A. Fornander M. J. (2019). Reconciling contemporary approaches to school attendance and school absenteeism: toward promotion and nimble response, global policy review and implementation, and future adaptability (part 1). Front. Psychol. 10:2222. doi: 10.3389/fpsyg.2019.02222 31681069 PMC6805702

[ref33] Kearney C. A. Graczyk P. A. (2014). A response to intervention model to promote school attendance and decrease school absenteeism. Child Care Q. 43, 1–25. doi: 10.1007/s10566-013-9222-1

[ref34] Kearney C. Graczyk P. (2020). A multidimensional, multi-tiered systems of support model to promote school attendance and address school absenteeism. Clin. Child. Fam. Psychol. Rev. 23, 316–337. doi: 10.1007/s10567-020-00317-1, PMID: 32274598

[ref35] King N. J. Heyne D. Tonge B. J. Mullen P. E. Myerson N. Rollings S.. (2003). Sexually abused children suffering from post-traumatic stress disorder: assessment and treatment strategies. Cogn. Behav. Ther. 32, 2–12. doi: 10.1080/16506070310003620, PMID: 16291530

[ref36] Laan A. M. Veenstra R. Veenstra R. Bogaerts S. Bogaerts S. Verhulst F. C.. (2010). Serious, minor, and non-delinquents in early adolescence: the impact of cumulative risk and Promotive factors. The TRAILS study. J. Abnorm. Child Psychol. 38, 339–351. doi: 10.1007/s10802-009-9368-3, PMID: 19957027 PMC2839520

[ref37] Lary M. Allsopp L. Lary D. Sterling D. (2019). Using machine learning to examine the relationship between asthma and absenteeism. Environ. Monit. Assess. 191, 1–9. doi: 10.1007/s10661-019-7423-2 31254081

[ref07] Li W. Bhutto T. A. Xuhui W. Maitlo Q. Zafar A. U. Bhutto N. A. (2020). Unlocking employees’ green creativity: the effects of green transformational leadership, green intrinsic, and extrinsic motivation. J. Cleaner Production 255:120229.

[ref38] McLeod S. (2018). B.F. skinner—operant conditioning. Available at: https://www.simplypsychology.org/operant-conditioning.html (Accessed April 3, 2022).

[ref39] Mervilde J. (1981). Student absenteeism: Causes, effects, and possible solutions. Available at: https://eric.ed.gov/?id=ed207157 (Accessed April 3, 2022).

[ref40] Mishra R. Trivedi P. (2011). Student attendance system based on fingerprint recognition and one to many matching. Available at: http://ethesis.nitrkl.ac.in/2214/1/thesis.pdf (Accessed April 3, 2022).

[ref41] Mitchell T. (1997). Machine Learning, Vol. 1. New York, NY: McGraw-hill.

[ref42] Moscoso R. Y. (2000). The effects of school characteristics on student academic performance. Available at: https://vtechworks.lib.vt.edu/handle/10919/27346 (Accessed April 3, 2022).

[ref43] Mushtaq I. Khan S. N. (2012). Factors affecting Students' academic performance. Glob. J. Manag. Bus. Res. 12, 17–22.

[ref44] National Center for Education Statistics (NCES) (2020). Improving MTSS/RTI implementation through measurement. US Department of Education. Washington DC: NCES. Available at: https://ies.ed.gov/ncee/edlabs/regions/appalachia/blogs/blog26_improving-MTSS-RTI-through-measurement.asp (Accessed March 2020).

[ref45] Ng H. Azha A. B. Yap T. Goh V. (2021). A machine learning approach to predictive Modelling of student performance. F1000 Res. 10:1144. doi: 10.12688/f1000research.73180.1, PMID: 35719314 PMC9194521

[ref46] Ng V. V. Heyne D. Kueh Y. C. Husain M. (2019). The Malay self-efficacy questionnaire for school situations: development, reliability, and validity among early adolescents in primary school. Eur. J. Educ. Psychol. 12, 91–108.

[ref47] Ngo F. T. Paternoster R. Cullen F. T. MacKenzie D. L. (2011). Life domains and crime: a test of Agnew's general theory of crime and delinquency. J. Crim. Just. 39, 302–311. doi: 10.1016/j.jcrimjus.2011.03.006, PMID: 35165158

[ref48] Parrish P. Linder-VanBerschot J. A. (2010). Cultural dimensions of learning: addressing the challenges of multicultural instruction. Int. Rev. Res. Open Dist. Learn. 11, 1–19. doi: 10.19173/irrodl.v11i2.809

[ref09] Roesch M. Singer C. (2013). Columbia University Work Life: Pros and Cons of Standardized Testing. Available at: https://www.everettsd.org/cms/lib07/WA01920133/Centricity/Domain/2847/Pros%20and%20Cons%20of%20Standardized%20Testing.pdf (Accessed October 15, 2022).

[ref50] Randle L. (1997). What is the effect of pre-school attendance on Reading achievement at the third grade? Available at: https://eric.ed.gov/?id=ed404630 (Accessed April 3, 2022).

[ref51] Rasasingham R. (2015). The risk and protective factors of school absenteeism. Psychiatry 5, 195–203. doi: 10.4236/ojpsych.2015.52023

[ref011] Rastrollo-Guerrero J. L. Gómez-Pulido J. A. Durán-Domínguez A. (2020). Analyzing and predicting students’ performance by means of machine learning: a review. Applied Sciences 10:1042.

[ref52] Reid K. (2005). The causes, views and traits of school absenteeism and truancy: an analytical review. Res. Educ. 74, 59–82. doi: 10.7227/RIE.74.6

[ref53] Roh M. Cho C. A. N. B. Kim J. J K., & & Gentle-Genitty C. (2022). The impact of life domains on delinquent behaviors in five Caribbean countries: a partial test of Agnew's general theory of crime and delinquency. Violence Vict. 37, 3–25. doi: 10.1891/VV-D-20-00206, PMID: 35165158

[ref54] Roh M. Marshall I. H. (2018). A cross-cultural analysis of Agnew’s general theory of crime and delinquency. Available at: http://sshj.in/index.php/sshj/article/download/85/51 (Accessed May 25, 2022).

[ref55] Rothman S. (2001). School absence and student background factors: a multilevel analysis. Int. Educ. J. 2, 59–68.

[ref56] Rozario R. R. Manjunatha H. (2018). Automatic face tracking and attendance system using machine learning techniques. Int. J. Tomogr. Simul. 31, 57–69.

[ref57] Sheldon S. B. Epstein J. L. (2004). Getting students to school: using family and community involvement to reduce chronic absenteeism. Sch. Community J. 14, 39–56.

[ref58] Skedgell K. Kearney C. A. (2016). Predictors of absenteeism severity in truant youth: a dimensional and categorical analysis. Am. Second. Educ. 45, 46–58.

[ref59] Slade E. P. Wissow L. S. (2007). The influence of childhood maltreatment on adolescents' academic performance. Econ. Educ. Rev. 26, 604–614. doi: 10.1016/j.econedurev.2006.10.003, PMID: 18037979 PMC2083567

[ref60] Sun R. C. Shek D. T. (2012). Classroom misbehavior in the eyes of students: a qualitative study. Sci. World J. 2012:398482. doi: 10.1100/2012/607157, PMID: 22919316 PMC3415076

[ref61] Suparwito H. (2019). Factors influencing the difficulty level of the subject: Machine learning technique approaches. Available at: https://e-journal.usd.ac.id/index.php/ijasst/article/view/1869/1498 (Accessed March 4, 2022).

[ref010] Taylor R. Oberle E. Durlak J. Weissberg R. (2017). Promoting positive youth development through school-based social and emotional learning interventions: a meta-analysis of follow-up effects. Society for Research in Child Development 88, 1156–1171. doi: 10.1111/cdev.12864 28685826

[ref62] Teaching and Learning Cognitivism (2022). Available at: http://teachinglearningresources.pbworks.com/w/page/31012664/Cognitivism (Accessed March 4, 2022).

[ref63] Toprceanu A. (2017). Gamified learning. Proc. Comp. Sci. 112, 41–50. doi: 10.1016/j.procs.2017.08.017, PMID: 36337566

[ref64] Tuan H.-L. Chin C.-C. Shieh S.-H. (2005). The development of a questionnaire to measure students' motivation towards science learning. Int. J. Sci. Educ. 27, 639–654. doi: 10.1080/0950069042000323737

[ref65] Vilsaint C. L. Aiyer S. M. Wilson M. N. Shaw D. S. Dishion T. J. (2013). The ecology of early childhood risk: a canonical correlation analysis of Children’s adjustment, family, and community context in a high-risk sample. J. Prim. Prev. 34, 261–277. doi: 10.1007/s10935-013-0305-4, PMID: 23700232 PMC4749130

[ref012] Weissberg R. P. Cascarino J. (2013). Academic learning+social-emotional learning= national priority. Phi Delta Kappan 95, 8–13.

[ref66] Zhang M. (2003). Links between school absenteeism and child poverty. Past. Care Educ. 21, 10–17. doi: 10.1111/1468-0122.00249, PMID: 29784550

